# Analysis and Validation of Ultrasonic Probes in Liquid Level Monitoring Systems

**DOI:** 10.3390/s21041320

**Published:** 2021-02-12

**Authors:** Wanjia Gao, Wenyi Liu, Fei Li, Yanjun Hu

**Affiliations:** Key Laboratory of Instrumentation Science & Dynamic Measurement, Ministry of Education, North University of China, Taiyuan 030051, China; 18810577682@163.com (W.G.); 18834169656@163.com (F.L.); 18234125986@163.com (Y.H.)

**Keywords:** ultrasonic, liquid level monitoring, ultrasonic probe, reflection and transmission

## Abstract

Selecting and designing the optimum ultrasonic probe is vital for ultrasonic measurements and experiments. The amplitude of the emitted ultrasonic wave excitation signal as well as the diameter and the natural frequency of the probe seriously affect the validity of the probe results. In this paper, we analyze the significance of the key parameters of the ultrasonic probe theoretically. Further, an external fixed-point liquid level monitoring system was assembled according to the principle of ultrasonic reflection and transmission. On this experimental platform, we study the key parameters of the ultrasonic probe that affect the system evaluation through a simulation and experiment, and select the optimal sensor parameters for this experiment. The evaluations show that under the experimental conditions where the tested container is made of aluminum alloy and its wall thickness is 3 mm, the best results are obtained when the diameter of the ultrasonic sensor is 15 mm, the amplitude of the emitted excitation signal is ±15 V, and the frequency is 1 MHz. The results’ average deviation is less than ±0.22 V. The evaluations are consistent with the simulation results. This research can effectively monitor the liquid in the closed, ultra-thin-walled container, and can realize non-contact measurement. It provides an effective basis for the parameters selection and design of the ultrasonic probe in the ultrasonic-based experiments and tests.

## 1. Introduction

In the fields of traditional industrial control, aerospace, and aviation, it is essential for real-time monitoring and alarming of closed containers or pressure vessels’ liquid levels to be accurate [1,2]. Therefore, the research of liquid level sensors is particularly important.

For harsh investigation environments that have features such as high temperatures, a high pressure, and sealing, some detection methods require the sensor to directly contact the measured liquid or to be installed with holes, which will damage the integrity of the tested container [3,4,5]. Non-contact and non-holes techniques in this context are the most appropriate measurement method [6,7]. Ultrasonic methods achieve a true sense of non-contact [8,9,10]. Ultrasonic sensors have been applied in the field of non-destructive testing. Zhang, M et al. [11] designed an untouched liquid level measurement system based on ultrasonics. They designed the system based on the principle of ultrasonic interface reflection, with sensors installed on the bottom of the measured container. This method is greatly affected by the temperature of the medium and the bubbles or impurities in the medium. Tat Hean Gan et al. [12] designed a non-contact ultrasonic system using an electrostatic transducer and signal processing technology, which has been applied in food detection. They also used the principle of ultrasonic transmission. The attenuation characteristics of ultrasonic waves affect the reliability of the measurement. It is not suitable for large containers. Zainal Zakaria et al. [13] developed a new method by using a non-invasive ultrasonic instrumentation system for monitoring the LPG (Liquefied Petroleum Gas) level in a 14-kg cylinder. Their design was based on the ultrasonic impedance method. But their system required multiple probe arrays, and the measurement error was 10%. Hao Haohao et al. [14] designed a liquid level monitoring system based on the ultrasonic impedance method. They monitored the liquid level by measuring the duration of echo energy decay based on the difference in reflectivity and transmittance caused by different levels of acoustic impedance between a gas and a liquid. The method required only one sensor and the design was simple. However, there were too many initial conditions to be determined in advance, and the threshold needed to be determined by repeated trials. This meant the method involved a complicated operation and low flexibility. For containers with ultra-thin walls, the measurement accuracy is also not high.

The detection of each of the parameters of the ultrasonic wave requires specific transducers and a dedicated electronic system for signal acquisition and parameter extraction. Many factors of ultrasonic sensors affect its evaluations [15]. Hao Haohao et al. [14] mentioned that the attenuation of sound increases with the frequency. They proposed that it is suitable if the frequency remains at around 1 MHz. Z. Yanjun et al. [16] conducted experiments using two ultrasonic probes with different diameters. They explored the influence of different transmitted wave voltage amplitudes and different container wall thicknesses on the ultrasonic echo sound pressure. Finally, it was concluded that the choice of probe diameter depends on the measurement of physical properties such as wall thickness. Danilov, V.N. [17] proposed that a decrease in the wear-plate thickness leads to an increase in the operating frequency. This circumstance can be used during selection of the parameters of a transducer for immersion testing. Lanoye, R. et al. [18] investigated a new method for the measurement of the surface impedance in the free field of a layer of absorbing material. They conducted comparative experiments and made a detailed analysis of the influence of the calibration, the source type, the source height, the sound incidence angle, and the sample size. Chen L et al. [19] developed an ultrasonic instrument for the sealed container liquid level measurement. They also adopted a line focusing intersected transducer with a focal length of 5 mm, which is the same as the wall thickness of the container.

Additionally, the ultrasound probe emits a beam of ultrasound that exists near the field. In the near-field area, the sound pressure changes irregularly, which causes the inaccuracy of the test results [20]. Therefore, the front buffer block of the ultrasonic sensor is also a key factor. In the previous research, our team studied the effect of different buffer block lengths on the evaluations in the ultrasonic-based liquid level detection system under the same other conditions [21]. The conclusion can effectively avoid the near-field area and provide a basis for the design of the ultrasonic probe.

To sum up, this paper firstly analyzes the relative parameters of the ultrasonic probe theoretically. On this theoretical basis, according to the principle of ultrasonic reflection and transmission, we built an ultrasonic liquid-level monitoring system. The internal medium can be distinguished by measuring the echo energy values of the container wall, which plays a liquid level monitoring role. Then we conducted experiments on a 3 mm-thick aluminum alloy container, and we analyzed the effects of essential parameters such as the transmitting amplitude, sensor diameter, and natural frequency on the ultrasonic echo energy. The evaluations determined the feasibility of the system, and can help select the most suitable sensor parameters. In addition, we used simulation software to establish a model and simulate the piezoelectric ceramics in the air domain, and used its total sound pressure field to help select the frequency. The system built in this paper can effectively monitor the liquid in the closed ultra-thin-walled container and realize non-contact measurement. The conclusions of this study provide an effective basis for the selection of ultrasonic transducer parameters in ultrasonic experiments.

## 2. Theory and Methods

### 2.1. Reflection and Transmission of Ultrasonic Waves

The experiments in this paper are based on the reflection and transmission characteristics of ultrasonic waves. The reflection and transmission of ultrasonic waves at different interfaces are closely related to the acoustic impedance of the two media. The acoustic impedance is equal to the product of the density of the medium and the wave velocity, as shown in Equation (1) [15]

(1) $$Z=\frac{P}{u}=\frac{\rho cu}{u}=\rho c$$

where *P* is the sound pressure, Pa; *u* is the particle vibration velocity, m/s; ρ is medium density, kg/m^3^; *c* is the medium sound velocity, m/s. Acoustic impedance is an important physical quantity to characterize the acoustic properties of media [22].

In ultrasonic testing, the echo height of the reflector displayed on the oscilloscope is only proportional to the reflected sound pressure, that is [23]:

(2) $$\frac{P_{1}}{P_{2}}=\frac{H_{1}}{H_{2}}.$$

When ultrasonic waves are perpendicularly located on two media with different acoustic impedances, the reflected wave returns in a path opposite to the incident wave, and the rest of the ultrasonic waves penetrate the interface and enter the second medium. According to the principle of continuous sound pressure and continuous vibration velocity on the interface, the reflection coefficient and transmission coefficient of sound pressure can be obtained by Equations (3) and (4) [24]

(3) $$\tau_{p}=\frac{P_{t}}{P_{0}}=\frac{2Z_{2}}{Z_{1}+Z_{2}}$$

(4) $$\gamma_{P}=\frac{P_{r}}{P_{0}}=\frac{Z_{2}-Z_{1}}{Z_{1}+Z_{2}}$$

where *P*_0_ is the incident sound pressure; *P_r_* is the reflected sound pressure; *P_t_* is the transmitted sound pressure. *Z*_1_ is the acoustic impedance of the medium on the incident side; *Z*_2_ is the acoustic impedance of the medium on the transmission side.

The experimental platform in this paper is to install the ultrasonic probe vertically on the outer wall of the aluminum alloy container, and compare the reflected echo energy received from the aluminum alloy-water and aluminum alloy-air interfaces. The acoustic impedance of relevant materials in the experiment is shown in Table 1 [21].

**Figure 1:**
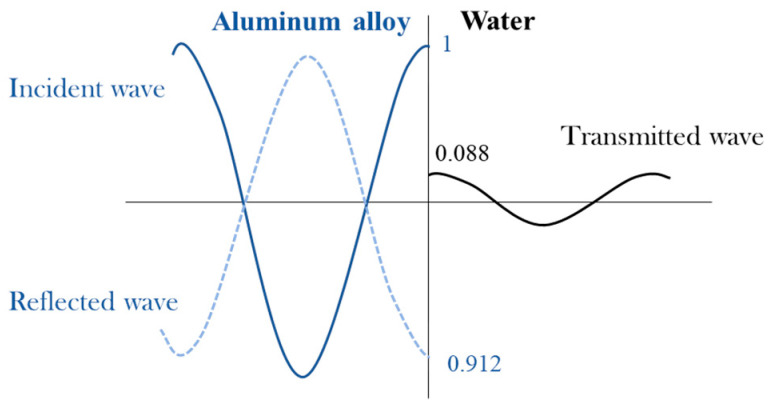
Sound pressure distribution of ultrasonic incident on the aluminum alloy-water interface.

Ultrasonic waves enter the water from the aluminum alloy, at this time *Z*_1_ > *Z_W_*, substituting *Z_1_* and *Z_W_* in Table 1 into Equations (3) and (4) to obtain the reflection coefficient $\gamma_{P1}=-0.912$, and the transmission coefficient $\tau_{p1}=0.088$. The negative sign indicates that the phase difference between the incident sound wave and the reflected sound wave is 180°. Figure 1 shows the sound pressure distribution of the above process.

It can be seen from Figure 1 that when incident waves are incident in a medium with large acoustic impedance to a medium with small acoustic impedance, the absolute value of reflected sound pressure is less than the incident sound pressure, while the phases of the two are opposite and cancel each other out. So the transmitted sound pressure value is extremely small.

Ultrasonic waves enter the air from the aluminum alloy, at this time *Z*_1_ >> *Z_A_*, substituting *Z*_1_ and *Z_A_* in Table 1 into Equations (3) and (4) to obtain the reflection coefficient $\gamma_{P2}\approx -1$, and the transmission coefficient $\tau_{p2}=0$. This shows that ultrasonic waves are totally reflected at the aluminum alloy-air interface without transmitting. Therefore, based on this principle, we can build a liquid level monitoring system outside the container.

### 2.2. Design of Liquid Level Monitoring System

**Figure 2:**
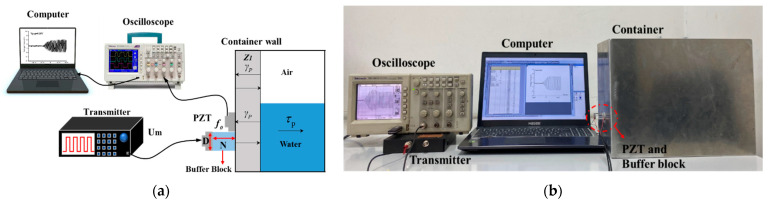
(**a**) Designed diagram of experimental platform; (**b**) Devices photo. (PZT = Piezoelectric ceramic; $f_{0}$ = Natural frequency of the sensors; *Um* = Amplitude of emitted excitation signal; *D* = Diameter of the sensors; *N* = Near-field length of the ultrasonic beam).

Based on the principle of ultrasonic impedance method, we designed an external fixed-point liquid-level monitoring experimental system. The system includes two sensors: one is used as the transmitter and the other is used as the receiver. Both sensors are piezoelectric ceramic (PZT) chips. A certain length of polymethyl methacrylate (PMMA) rod is added between the transmitter sensor and the container wall as a buffer block, so that the emitted ultrasonic wave reaches the container wall in the far-field area. We installed them on the outside of the container wall, and made the sensor perpendicular to the container wall. The receiver PZT was installed next to the transmitter and was at the same height. We filled the gap with a medical couplant so that the air could be expelled and as much ultrasonic energy as possible could be transmitted into the container wall. We received the remaining energy reflected from the inner wall of the container, collected the data by oscilloscope (TDS 1001B, Tektronix, Shanghai, China), and then processed the data by using a computer. Through the echo energy value, it was possible to determine whether the internal medium at the height of the sensor was gas or liquid. The designed diagram and the devices photo are shown in Figure 2.

In this experiment, the tested container was made of aluminum alloy. The wall thickness is 3 mm. The interior of the container was trapezoidal. Sensors were installed on one side perpendicular to the ground, and the opposite side was inclined at 45°. In this way, the receiver received less ultrasonic echo reflected from the opposite container wall, which reduced interference and improved the accuracy of the experiment. The medium inside the container was air and water. The experiment was carried out at a constant temperature of 20 °C to keep the speed of sound constant.

In this experiment, the ultrasonic echo energy was converted into electrical signals, and the peak-to-peak value of waveform read by oscilloscope was taken as the measured value of the experiment. According to the theoretical analysis in Section 2.1, water has a higher transmission ability than air, and the type of medium can be judged based on the amplitude of the received echo signal.

### 2.3. Selection of Ultrasonic Probe

The ultrasonic probe used in this experiment is made of piezoelectric wafers. Some important parameters of the probe affect the emitting ultrasonic wave energy. The product of the thickness of the piezoelectric wafer and the natural frequency is a constant [25,26]. With the same material, the wafer thickness is smaller when making the high-frequency probes. When making low-frequency probes, the wafer thickness is relatively large. The frequency of the emitted ultrasonic wave depends mainly on the thickness of the wafer and the sound velocity in the wafer.

There is a near-field area in the sound field near the wave source, denoted by *N*, which satisfies Equation (5) [27]:

(5) $$N=\frac{D_{s}^{2}}{4\lambda }$$

where *D_s_* is the wafer diameter, m; λ is the acoustic wavelength, m. A beam of ultrasonic waves emitted by the ultrasonic probe has a non-diffused area near the wave source for a certain distance, energy is concentrated in this area. Then the ultrasonic waves diffuse at a certain angle [28].

The ultrasonic wave energy attenuates with the increase of distance, and its scattering attenuation coefficient is $\alpha_{S}$, which meets the requirements of Equation (6) [29]:

(6) $$\alpha_{S}=c_{2}Fd^{3}f^{4}(d<\lambda )$$

where *c_2_* is a constant; *F* is the anisotropy coefficient; *d* is the grain diameter of the medium; *f* is the acoustic frequency.

According to the above theoretical analysis, the appropriate ultrasonic probe should be selected first for different testing experiments.

Frequency is an important parameter of an ultrasonic testing instrument. The frequency of ultrasonic testing is between 0.5–10 MHz. According to [28], the frequency is high, the wavelength is short, the semi-diffusion angle is small, the sound beam has good directivity, and the energy is concentrated. According to Equation (5), the higher the frequency, the shorter the wavelength and the larger the length of the near field, which is unfavorable for detection. It can be seen from Equation (6) that the frequency increases and the attenuation increases sharply. According to [25,26], the lower the frequency, the thicker the wafer thickness. Therefore, the frequency has a great influence on detection.

The size of the probe chip also affects detection [30]. Its diameter is generally 10–30 mm. According to [28], the wafer size increases, the semi-diffusion angle decreases, and the ultrasonic energy concentrates. It can be seen from Equation (5) that the diameter of the chip increases and the length of the near field increases. According to [31], the sound pressure emitted by the chip to the measured object is proportional to the square of the diameter of the chip. To balance the test sensitivity and resolution of the probes in the experiment, we conducted experimental research on probes with different diameters.

According to Equation (2), the voltage amplitude of the emitted excitation signal affects the sound pressure of the reflected wave. Therefore, the amplitude of the emitted ultrasonic wave excitation signal is also a key factor. In this paper, we studied the different amplitude of the emitted excitation signal experimentally.

The relationship between the near field length and PZT diameter has been studied in the previous paper published by our team [21]. Therefore, we directly selected the optimal length of the buffer block for experimentation based on the PZT diameter for the current study.

## 3. Results and Discussion

To explore the influence of sensor diameter, natural frequency, and wave excitation signal amplitude on the whole experiment, we conducted experiments in groups on the experimental platform built in this article. We installed the ultrasonic sensor at the same height outside the container, and we could determine the working efficiency of the sensor according to the difference in the amplitude of the received ultrasonic echo signal. The specifications of the PZT plates used in the experiment are shown in Table 2. Three kinds of excitation signal amplitude: ±5 V, ±10 V, and ±15 V, were selected for relevant experiments.

### 3.1. Sensor Diameter Analysis

**Figure 3:**
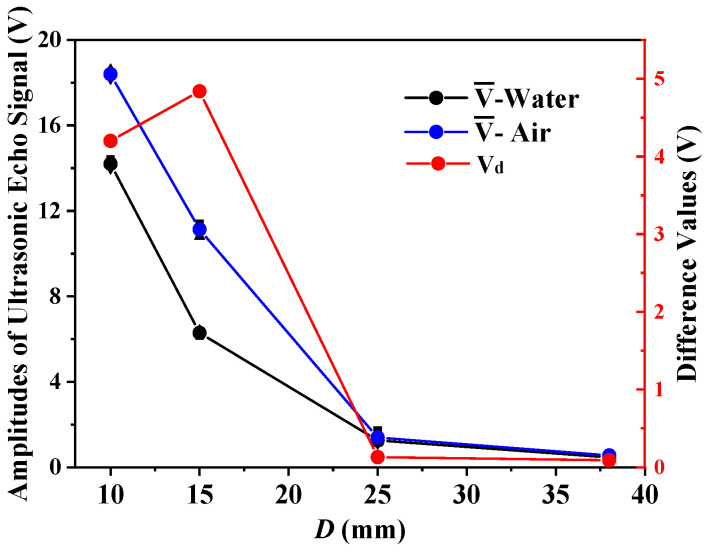
Results comparison diagram with different diameters.

From Table 2, the PZT plates with a frequency of 1 MHz and diameters of 10 mm, 15 mm, 25 mm, and 38 mm were selected for the first set of experiments. The amplitude of the emitted excitation signal was ±15 V. The experiment was conducted under the same conditions to explore the impact of different diameters on the ultrasonic probe, and the experiments were performed three times in each group. We recorded the amplitude of the echo signal received by the sensor, and calculated the average values $\bar{V}$, the average deviations |ΔE|, and the difference values V_d_. The evaluations are shown in Table 2. Figure 3 shows the contents of Table 3.

It can be seen from the evaluations in Table 3 and Figure 3 that under the condition of the same natural frequency and transmitting wave voltage, the experimental effects of PZT plates with 10 mm and 15 mm diameters are obvious. When the diameter is too large, the difference V_d_ of the echo signal amplitude received by the sensor at the water and air is small, and the experimental effect is poor. It is difficult to distinguish the liquid level above or below the sensor. This is because when the nature frequency of the sensor is the same, the increase in diameter leads to an increase in the near-field area of the transmitted wave. Therefore, the length of the buffer block between the probe and the container increases, and more energy loss occurs in the process of ultrasonic transmission. Although the diffusion angle decreases and the acoustic beam has better directivity, when the diameter of the probe is too large, its lateral resolution decreases.

Therefore, it can be concluded that large diameter PZT plates are not suitable for measuring the liquid level in the ultra-thin-wall container by the ultrasonic impedance method. As can be seen from Table 3, the average deviations of multiple measurements are less than 0.22 V. As can also be seen from the echo signal difference values, the 15 mm PZT plate has a more obvious experimental effect in this experiment, and its measurement accuracy and sensitivity are higher.

### 3.2. Sensor Natural Frequency Analysis

To aid with understanding of the process of ultrasonic propagation, the sound field was visualized to obtain the optimal natural frequency of the sensor required by the experiment. To achieve this, we built a finite element simulation model based on COMSOL and compared the simulation results. The model uses frequency domain analysis to simulate the distribution of ultrasonic waves generated by PZT in an infinite air domain.

**Figure 4:**
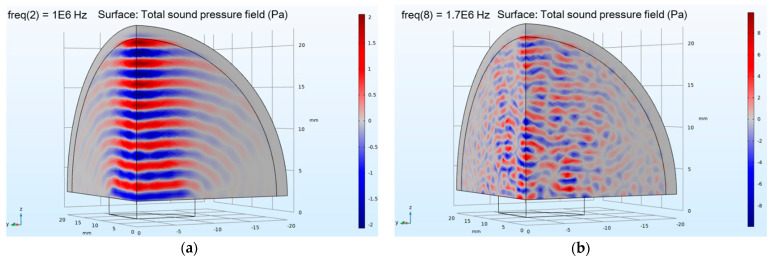
The total sound pressure field: (**a**) the resonant frequency of the transducer is 1 MHz; (**b**) the resonant frequency is 1.7 MHz.

The model refers to the actual parameters of the PZT used in the experiment, while the cylinder as the sensor was set to 15 mm in diameter and 2 mm in thickness. A voltage driving signal of 15 V was loaded on its boundary to excite ultrasonic waves. The hemispherical air domain with a diameter of 40 mm was adjacent to the PZT, and its sound velocity c was 340 m/s. The outermost layer of the air domain was set as a perfectly matched layer with the same sound velocity to absorb sound waves and avoid boundary reflection. To save computation time and computer memory, only one quarter of the 3D model was reserved for simulation calculation. The simulation analyzed the sound field distribution in the frequency range of 200 kHz to 2 MHz. The total sound pressure field with frequencies of 1 MHz and 1.7 MHz are shown in Figure 4a,b, respectively.

Figure 4a,b show the simulation results of the total ultrasonic sound pressure field of the PZT in the air domain. In the figure, red is the peak and blue is the trough. The darker the red or blue, the greater the sound pressure. The higher the frequency, the shorter the wavelength, so the red and blue distribution becomes denser. As can be seen from Figure 4a, when the frequency is 1 MHz, the sound field distribution of ultrasonic waves propagating in the air domain is clear. The sound wave propagates regularly, and the sound pressure is evenly distributed, which proves that the sound waves propagation effect is good at this frequency. As can be seen from Figure 4b, when the frequency is increased to 1.7 MHz, the peak and trough of the wave are superimposed, and the distribution of sound waves in the air domain is irregular. Sound waves travel poorly at this frequency. When the frequency is low, the sound pressure is small, which is not conducive to the experiment. Based on the simulation results, it can guide the selection of probe frequency.

**Figure 5:**
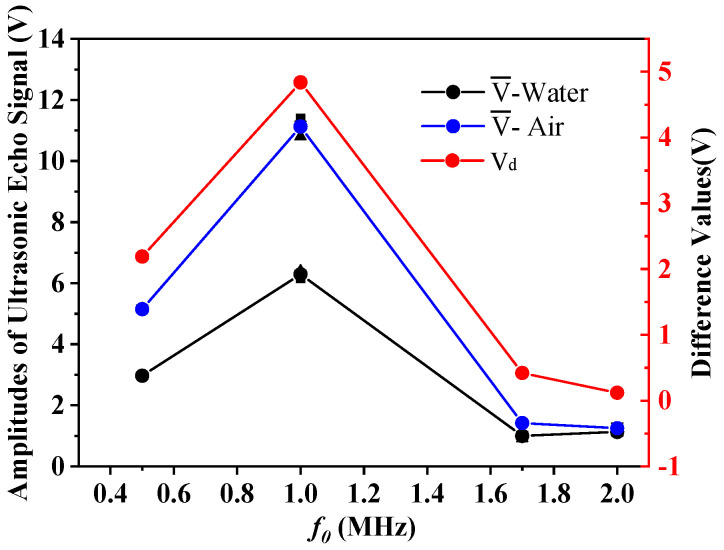
Results comparison diagram with different frequencies.

The PZT plates with a diameter of 15 mm and natural frequencies of 500 kHz, 1 MHz, 1.7 MHz, and 2 MHz in Table 2 were selected for the second set of experiments, and the amplitude of the emitted excitation signal was ±15 V. The experiments were performed three times in each group. We recorded the amplitude of the echo signal received by the sensor, and calculated the average values $\bar{V}$, the average deviations |ΔE| and the difference values V_d_. The evaluations are shown in Table 4. Figure 5 shows the contents of Table 4.

**Figure 6:**
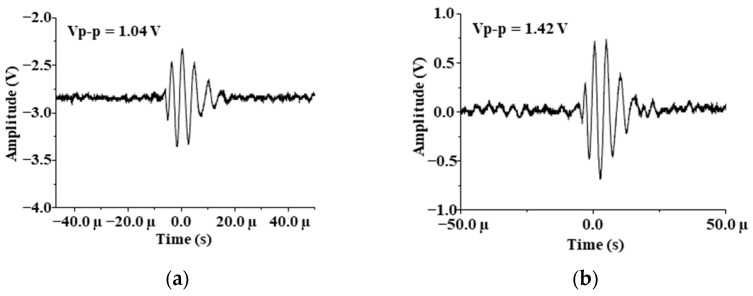
Echo images drawn by computer: (**a**) For water in 1.7 MHz; (**b**) For air in 1.7 MHz.

From Table 4 and Figure 5, it can be seen that when the frequency is 1.7 MHz and 2 MHz, the received signal energy is weak, and the amplitude difference V_d_ between water and air is also small, which cannot meet the demands of liquid level monitoring. When the frequency is 500 kHz and 1 MHz, the results are reasonable. This is because the nature frequency increases, the diffusion angle decreases, and the wave energy is more concentrated. However, as the frequency increases, the length of the near field increases, and the energy attenuation increases sharply. Therefore, it can be seen from the results in Figure 6 that when using the PZT with a natural frequency of 1 MHz, the results obtained are better than those obtained at other frequencies. It is most appropriate to keep the frequency at 1 MHz. As can be seen from Table 4, the average deviations of multiple measurements are less than 0.22 V. The experimental conclusion is consistent with the simulation results in Figure 4, which proves the reliability of the experiment. When the frequency is 1.7 MHz, the data received by oscilloscope is imported into the computer and the echo images are drawn as shown in Figure 6.

### 3.3. Emitted Excitation Signal Amplitude Analysis

**Figure 7:**
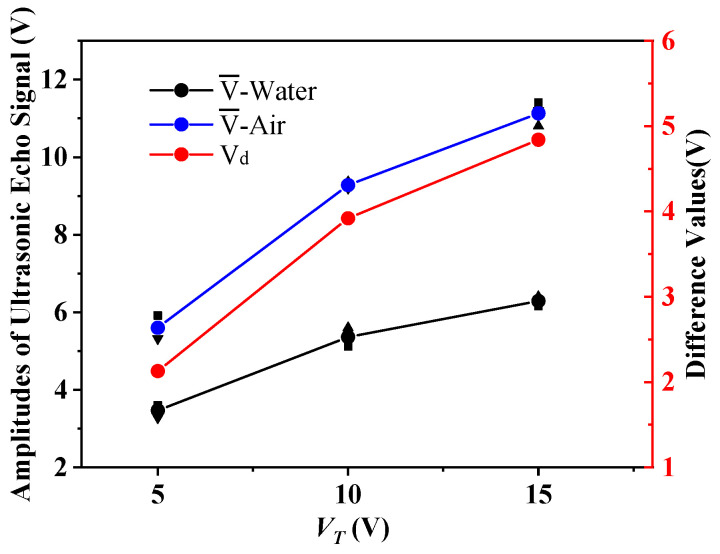
Results comparison diagram with different amplitudes.

The PZT plate with a diameter of 15 mm and a natural frequency of 1 MHz was selected for the third set of experiments, and the amplitude of the emitted excitation signal V_T_ was set to ±5 V, ±10 V, and ±15 V, respectively. The experiment was conducted under the same conditions to explore the influence of different amplitudes on evaluations, and the experiments were performed three times in each group. We recorded the amplitude of the echo signal received by the sensor, and calculated the average values $\bar{V}$, the average deviations |ΔE|, and the difference values V_d_. The evaluations are shown in Table 5. Figure 7 shows the contents of Table 5.

From the comparison of the data in Table 5 and Figure 7, it can be seen that the three groups of experiments all have good results. There are apparent differences in the echo signal amplitude received by the sensor. The larger the voltage, the more significant the echo difference. All three sets of experiments can effectively detect the type of medium inside the container. As can be seen from Table 5, the average deviations of multiple measurements are less than 0.22 V.

It can be seen from Figure 7 that the experimental results do not show good linearity, and the reasons are analyzed as follows. Temperature has a large influence on sound velocity, and there may be deviations in the temperature during the experiment. The amount of coupling agent between the probe and the device causes certain errors in the experimental results. There may be some signal interference in the circuit of receiving and transmitting ultrasonic waves. Artificial readings of experimental data cause some errors. However, the results in Figure 7 are basically linear. The three ultrasonic excitation voltage values can meet the experimental requirements, and the experimental rule is consistent with the theory, which ensures the reliability of the experiments.

**Figure 8:**
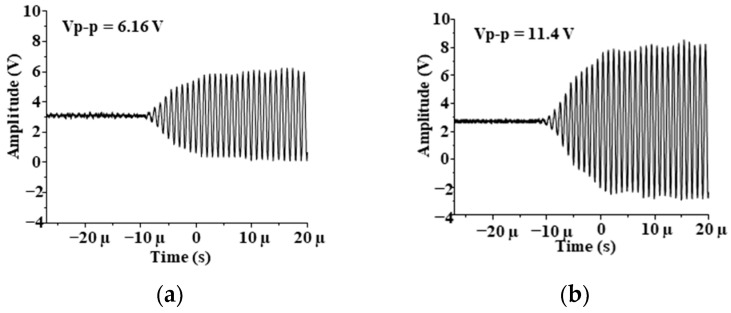
Echo images drawn by computer: (**a**) For water; (**b**) For air.

According to the experiments and analysis of the impact of the above three factors on the sensor, for the closed aluminum alloy container with a wall thickness of 3 mm, the sensor optimal parameters for the liquid-level monitoring design are: diameter = 15 mm; natural frequency = 1 MHz; emitted excitation signal amplitude = ±15 V. The waveform image data received by oscilloscope was imported into the computer, and the echo images are shown in Figure 8.

When the liquid level in the container is higher than the sensor, the waveform received by the receiver is shown in Figure 8a. When the liquid level is lower than the sensor, the waveform is shown in Figure 8b. When the liquid level is near the sensor, the waveform moves up and down. By comparing the amplitude change of the signal waveform, we can determine whether the liquid level in the closed container is higher or lower than the sensor. Installing the sensor at the position that needs to be monitored can play a role in liquid level monitoring or alarms.

## 4. Conclusions

This paper built a non-contact liquid level monitoring system based on the ultrasonic transmission and reflection principle. This method can monitor the liquid level in a closed container with an ultra-thin wall thickness (3 mm) in real time. Then, we analyzed the influence of the key parameters of the ultrasonic sensor on the results. At the same time, a model of a 15 mm diameter PZT in an infinite air domain was simulated by COMSOL, as well as its total sound pressure field with the frequency range of 200 kHz to 2 MHz. According to the analysis, the diameter and natural frequency of the probe, and the amplitude of the excitation signal affect the experimental results. The simulation results show that the sound field distribution is the most regular when the frequency is around 1 MHz and the sound waves propagation effect at its best level. Further, we conducted corresponding experiments to prove the conclusions. The evaluations show that under the situation of 20 ℃, the optimal parameters of the sensor obtained by the comprehensive test are: the diameter is 15 mm, the natural frequency is 1 MHz, and the emitted excitation signal amplitude is ±15 V. When the internal medium is air and water respectively, the obtained difference voltage of ultrasonic echo reaches 4.84 V, and the error is less than ± 0.22 V. The experimental results agree well with the simulation data, which proves the effectiveness of this method. The system built in this paper can effectively monitor the liquid in a closed ultra-thin-walled container without destroying the integrity of the container. The theoretical analysis and conclusions of this paper provide an effective basis for design and selection of ultrasonic probes in ultrasonic experiments. In the application of non-destructive testing of liquid level, the research in this paper improves the safety and reliability of measuring equipment.

## Figures and Tables

**Table 1:** Acoustic impedances of different media.

*Z*_1_ (g/cm^2^·s)	*Z_W_* (g/cm^2^·s)	*Z_A_* (g/cm^2^·s)
32 × 10^5^	1.48 × 10^5^	40

*Z*_1_: Acoustic impedance of aluminum alloy; *Z_W_*: Acoustic impedance of water; *Z_A_*: Acoustic impedance of air.

**Table 2:** Experimental piezoelectric ceramic (PZT) plates specifications.

*D* (mm)	*f*_0_ (MHz)
10	1
15	0.5, 1, 1.7, 2
25	1
38	1

**Table 3:** Evaluations with different diameters.

*D* (mm)	Medium	V_1_ (V)	V_2_ (V)	V_3_ (V)	$\bar{V}$	|ΔE| (V)	V_d_ (V)
10	Water	14.4	14.2	14	14.2	0.13	4.2
	Air	18.4	18.6	18.2	18.4	0.13	
15	Water	6.16	6.40	6.32	6.29	0.09	4.84
	Air	11.40	10.8	11.20	11.13	0.22	
25	Water	1.52	1.14	1.12	1.26	0.17	0.13
	Air	1.72	1.24	1.2	1.39	0.22	
38	Water	0.432	0.504	0.448	0.46	0.03	0.09
	Air	0.648	0.528	0.488	0.55	0.06	

V_1_: The first measured voltage value; V_2_: The second measured voltage value; V_3_: The third measured voltage value; $\bar{V}$: The average values; |ΔE|: The average deviations; V_d_: The difference values.

**Table 4:** Evaluations with different frequencies.

*f*_0_ (MHz)	Medium	V_1_ (V)	V_2_ (V)	V_3_ (V)	$\bar{V}$	|ΔE| (V)	V_d_ (V)
0.5	Water	2.94	2.98	2.98	2.97	0.02	2.19
	Air	5.12	5.16	5.18	5.15	0.02	
1	Water	6.16	6.4	6.32	6.29	0.09	4.84
	Air	11.4	10.8	11.2	11.13	0.22	
1.7	Water	1.04	0.92	1.04	1.00	0.05	0.42
	Air	1.44	1.42	1.4	1.42	0.01	
2	Water	1.16	1.1	1.14	1.13	0.02	0.12
	Air	1.2	1.24	1.32	1.25	0.04	

**Table 5:** Evaluations with different amplitudes.

V_T_ (V)	Medium	V_1_ (V)	V_2_ (V)	V_3_ (V)	$\bar{V}$	|ΔE| (V)	V_d_ (V)
5	Water	3.60	3.52	3.28	3.47	0.12	2.13
	Air	5.92	5.56	5.32	5.60	0.21	
10	Water	5.12	5.60	5.36	5.36	0.16	3.92
	Air	9.28	9.36	9.20	9.28	0.05	
15	Water	6.16	6.40	6.32	6.29	0.09	4.84
	Air	11.40	10.80	11.20	11.13	0.22	

## Data Availability

Not applicable.
